# Catheter Ablation of Ventricular Arrhythmias Originating From the Region of DGCV-AIV via a Swartz Sheath Support Approach

**DOI:** 10.3389/fcvm.2021.801441

**Published:** 2021-12-23

**Authors:** Cheng Zheng, Wei-Qian Lin, Yao-Ji Wang, Fang-Zhou Lv, Qi-Qi Jin, Jin Li, Jia-Feng Lin

**Affiliations:** Department of Cardiology, The Second Affiliated Hospital and Yuying Children's Hospital of Wenzhou Medical University, Wenzhou, China

**Keywords:** distal great cardiac vein, anterior interventricular vein, summit-communicating vein, ventricular arrhythmias, radiofrequency catheter ablation, Swartz sheath

## Abstract

**Aims:** This study aimed to investigate an appropriate catheter manipulation approach for ventricular arrhythmias (VAs) originating from the left ventricular epicardium adjacent to the transitional area from the great cardiac vein to the anterior interventricular vein (DGCV-AIV).

**Methods:** A total of 123 patients with DGCV-AIV VAs were retrospectively analyzed. All these patients underwent routine mapping and ablation by conventional approach [Non-Swartz sheath support (NS) approach] firstly. In the situation of the distal portion of the coronary venous system (CVS) not being accessed or a good target site not being obtained, the Swartz sheath support (SS) approach was attempted alternatively. If this still failed, the hydrophilic coated guidewire and left coronary angiographic catheter-guided deep engagement of Swartz sheath in GCV to support ablation catheter was performed.

**Results:** A total of 103 VAs (103/123, 83.74%) were successfully eliminated in DGCV-AIV. By NS approach, the tip of the catheter reached DGCV in 39.84% VAs (49/123), reached target sites in 35.87% VAs (44/123), and achieved successful ablation in 30.89% VAs (38/123), which was significantly lower than by SS approach (88.61% (70/79), 84.81 % (67/79), and 75.95% (60/79), *P* < 0.05). There were no significant differences in complication occurrence between the NS approach and the SS approach (4/123, 3.25% vs. 7/79, 8.86%, *p* > 0.05). The angle between DGCV and AIV <83° indicated an inaccessible AIV by catheter tip with a predictive value of 94.5%. Width/height of coronary venous system>0.69 more favored a SS approach with a predictive value of 87%.

**Conclusion:** For radiofrequency catheter ablation (RFCA) of VAs arising from DGCV-AIV, the SS approach facilitates the catheter tip to achieve target sites and contributes to a successful ablation.

## Introduction

Radiofrequency catheter ablation (RFCA) is an effective and safe therapy for idiopathic ventricular arrhythmias (VAs). However, the ablation of VAs originating from the left ventricular epicardium adjacent to the transitional area from the great cardiac vein to the anterior interventricular vein (DGCV-AIV) can be challenging because of the complex anatomic structures of this region (adjacent to coronary arteries) and difficulty in manipulation of the ablation catheter in the small-lumen and tortuous coronary venous system (1, 2).

It is reasonable to assume that any approach which could assist ablation catheter going through the anatomic obstacles in the coronary venous system and aid catheter tip reaching more distal portion of DGCV-AIV would improve the success rate of RFCA. Nevertheless, up to now, no systemic studies investigated the most appropriate manipulation approach for RFCA of VAs arising from DGCV-AIV.

Recent studies revealed that application of the Swartz sheath could improve the stability of catheter manipulation and enhance mapping and ablation efficiency in RFCA for VAs, for example, reversed U-curve technique of ablation catheter with the support of Swartz sheath for PSCs VAs (3), reversed S-curve technique of ablation catheter with the support of Swartz sheath close to the fossa ovalis for endocardial LV summit VAs (4). Thus, we doubt whether the Swartz sheath support approach could be utilized in the RFCA of DGCV-AIV VAs to achieve a more efficient ablation.

In this study, we aimed to evaluate the value and safety of the Swartz sheath support approach in the mapping and ablation of DGCV-AIV VAs.

## Methods

### Study Population

A total of 2768 patients (mean age 49.07 ± 17.40 years) were referred for RFCA for symptomatic VAs in our center from December 2009 to December 2020. Among them, 123 consecutive VAs were confirmed arising from the DGCV-AIV based on systemic mapping results or successful ablation sites and enrolled in this retrospective study. All patients had a normal ECG during sinus rhythm. Complete physical examination, echocardiography, exercise stress testing, or coronary angiography proved no structural heart diseases in any patient. Ethical approval was obtained from the hospital's ethics committee, and all patients gave written informed consent before operation.

### Mapping and Ablation

Electrophysiological study and ablation were performed after discontinuation of all anti-arrhythmic drugs for at least five half-lives. A 6F decapolar catheter (4-mm interelectrode spacing) was inserted from the right internal jugular vein and placed in the coronary sinus as distal as possible. If clinical arrhythmias failed to occur spontaneously, intravenous isoproterenol infusion (2–5 mg/min) was administered. An irrigated-tip ablation catheter was advanced to the right ventricle via antegrade transvenous approach and to the left ventricle via retrograde aortic approach. During VAs, the RV endocardium, LV endocardium, and aortic cusps were mapped cautiously and compared with the distal bipolar electrodes of the coronary sinus catheter, in order to identify the earliest site of ventricular activation. If comprehensive mapping results showed the earliest activation sites were in the distal portion of the coronary venous system (CVS), we would consider the VAs as arising from DGCV-AIV. For DGCV-AIV, the irrigated tip was applied with a flow rate of 30–60 ml/min, preset power of 25–30 W. CAG was performed in all cases to investigate the distance from the catheter tip to adjacent coronary arteries before RFCA. Energy delivery was forbidden when the distance was less than 5 mm. Coronary blood supply was routinely evaluated before and after ablation. If VAs were terminated or accelerated during the initial 10 s, radiofrequency delivery would be continued for 60 to 180 s. Otherwise, other targets were sought.

After successful ablation, intravenous administration of isoproterenol and programmed stimulation were performed to induce clinical VA. Acute success was defined as both an absence of spontaneous or provoked clinical VA at the end of the procedure and the latter 48-h period post-ablation on ECG Holter.

### Swartz Sheath Support Approach and Non-Swartz Sheath Support Approach

**Figure 1:**
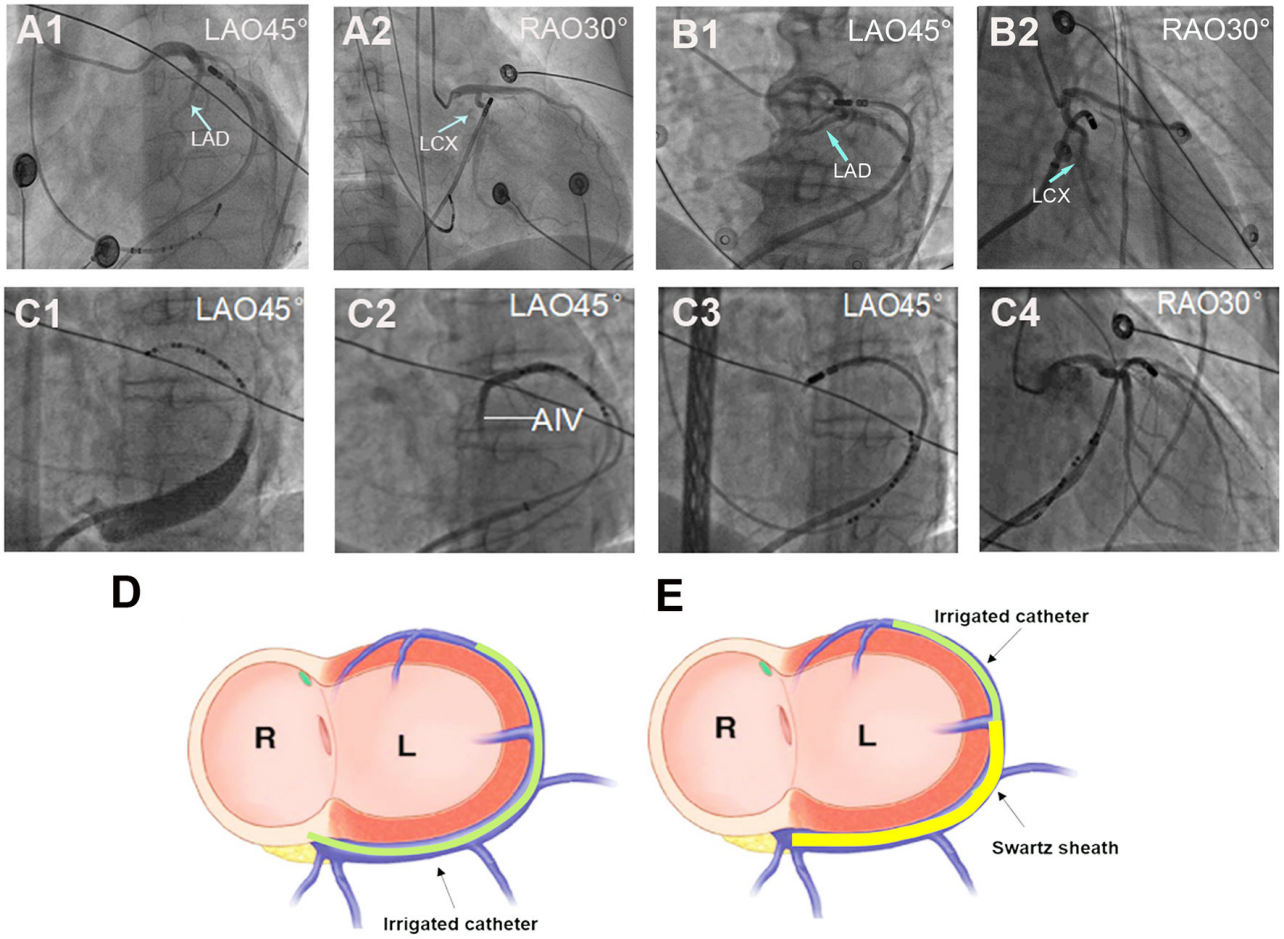
Different approaches for ablation catheter reaching target sites in DGCV. **(A)** Manipulation of the ablation catheter in GCV by non-Swartz sheath support (NS) approach. **(A1,A2)** The tip of the catheter was delivered to DGCV from the ostium of the coronary sinus directly. **(B)** Manipulation of the ablation catheter in GCV by Swartz sheath support (SS) approach. **(B1,B2)** The tip of the catheter was delivered to summit-CV with support from Swartz sheath in the GCV. **(C)** Super smooth guidewire and left coronary angiographic catheter-guided deep engagement of Swartz sheath in GCV. **(C1)** Due to the blockage of the Vieussens valve (shown by coronary venography), even with the support of Swartz sheath, the catheter tip could not reach the middle part of GCV. **(C2)** The hydrophilic coated guide wire and Judkin's 4-left coronary catheter were delivered through a Swartz sheath and passed the Vieussens valve and reached the DGCV. **(C3,C4)** The guided guide wire and coronary catheter, the Swartz sheath was advanced alternately and passed the Vieussens valve, then the guidewire and Jukin's catheter was exchanged with ablation catheter to perform mapping and ablation in DGCV. **(D)** Schema of NS approach for DGCV-AIV VAs. Targeting DGCV-AIV VAs via advancing ablation catheter from the ostium of coronary sinus directly. **(E)** Schema of SS approach for DGCV-AIV VAs. Targeting DGCV-AIV VAs via advancing ablation catheter from the ostium of the coronary sinus with the support from Swartz sheath. LAD, left anterior descending artery; LCX, left circumflex artery. LAO, left anterior oblique; RAO, right anterior oblique.

When the DGCV-AIV was considered the origin of ventricular arrhythmias, detailed mapping in DGCV-AIV was performed. For mapping and ablation in DGCV-AIV, two catheter manipulation approaches could be adopted, as shown in Figure 1. The conventional approach, the non-Swartz sheath support (NS) approach, was facilitated by delivering the tip of the ablation catheter directly from the ostium of the coronary sinus to DGCV-AIV to perform mapping and ablation. The Swartz sheath support (SS) approach was facilitated by engagement of Swartz sheath in GCV from the ostium of coronary sinus by ablation catheter. Then the ablation catheter and Swartz sheath were advanced alternately in GCV to reach the distal portion of DGCV-AIV to perform mapping and ablation. If the Swartz sheath still could not go through the GCV, the hydrophilic coated guide wire and left coronary angiographic catheter-guided deep engagement of Swartz sheath in GCV was conducted to support the ablation catheter.

### Definition of the Location of DGCV-AIV Origin

**Figure 2:**
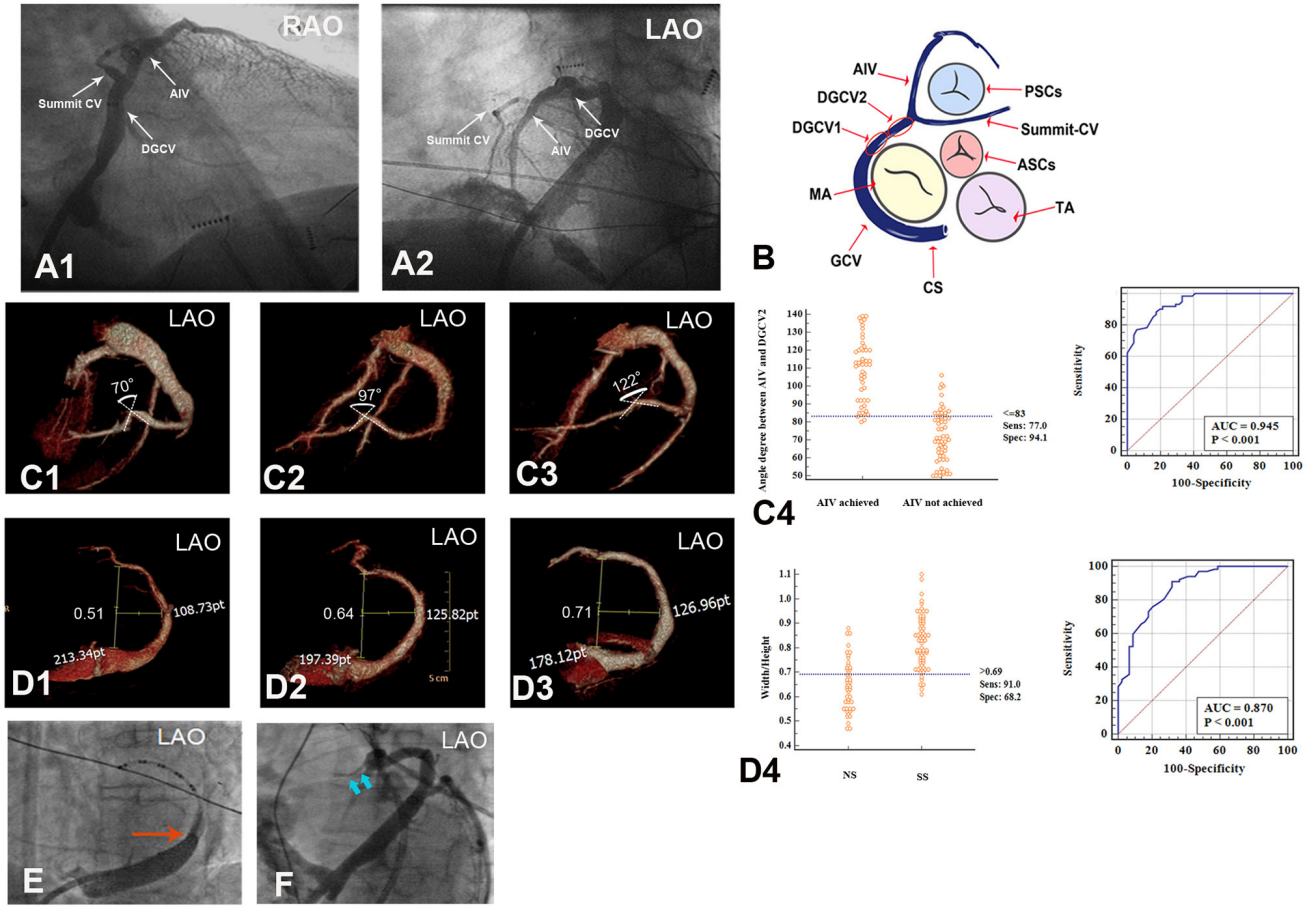
Anatomy of DGCV-AIV and anatomic obstacles preventing catheterization in GCV. **(A,B)** Anatomy of DGCV-AIV. **(C,D)** Anatomic obstacles preventing catheterization in GCV. **(D)** Angle formed between DGCV and AIV, and an angle <83° prevented the ablation catheter from advancing from DGCV into AIV. **(E)** Different morphology of GCV, and Width/Hight>0.69 more favored the application of the SS approach. **(E)** Venous valve (Vieussens valve) in GCV. **(F)** Thin lumen of summit-CV. DGCV, distal great cardiac vein; AIV, anterior interventricular vein; summit-CV, communicating vein in the left ventricular summit. RVOT, right ventricular outflow tract; LVOT, left ventricular outflow tract; TA, tricuspid annulus; MA, mitral annulus; CS, coronary sinus.

The DGCV-AIV origin of VAs was identified by mapping and ablation outcomes combined with retrograde venography of the coronary venous system. In our study, DGCV-AIV refers to the anatomy in the distal portion of the great cardiac vein, including four regions: DGCV1, DGCV2, summit-CV, and AIV. DGCV1 is defined as the segment of DGCV at the epicardium of the anterolateral wall of mitral annulus and DGCV2 as the segment of DGCV transecting the epicardial LV outflow region bounded by the bifurcation between the left anterior descending artery and left circumflex artery and continued to DGCV1. Anterior interventricular vein (AIV) refers to the vein going along the anterior interventricular groove from cardiac apex to bottom, turning posteriorly and continuing as DGCV2. Summit-CV refers to the communicating vein (CV) between the aortic and pulmonary annulus, which is the extended tributary of the DGCV located distal to the origin of the AIV, see Figure 2.

### Anatomic Obstacles for Catheter Manipulation in GCV

For catheter ablation of DGCV-AIV VAs, a thorough understanding of CVS anatomy is essential (1, 2). The coronary sinus is located at the posterior and inferior part of the epicardial mitral valve and, collecting the blood from the CVS, ends in the right atrium. There is a small folded tissue known as the Thebesian valve at the ostium of the coronary sinus, which might occasionally be an obstacle to catheterization. A small left atrial vein named Marshall (or Marshall ligament) is the remnant of the embryonic left superior cardinal vein and drains into the coronary sinus. It is at the point where the Marshall vein drains into the coronary sinus that the coronary sinus turns into the great cardiac vein, 29.15% of patients have a well-developed Vieussens valve at this site that might preclude ablation catheter advancement. The GCV goes along the lateral portions of the mitral valve and extends into DGCV at the epicardium of the anterolateral portion of the mitral annulus. It is reasonable to believe that a curved GCV morphology may limit the advancement of a catheter. DGCV turns into AIV beneath the aortic valve cusp at the left ventricular summit. The angle between AIV and DGCV2 has great individual variability. It is observed that an acute angle between AIV and DGCV2 would prevent the ablation catheter from reaching proximal AIV, on the contrary, an obtuse angle would facilitate catheter performing mapping and ablation in proximal AIV. Communicating vein refers to the very thin veins between the GCV and conus branch that drains to the small cardiac vein, and Summit-CV is a distinct CV that is located between the aortic and pulmonary annulus, distal to the transitional area between the GCV and the AIV, and in close association with the superior portion of the LV summit. Previous studies have revealed summit-CV can be the source of idiopathic ventricular arrhythmias. However, the very thin lumen of this vessel usually limits the detailed mapping and ablation in this region. Above all, hampering of venous valves (Thebesian valve and Vieussens valve), deflections of GCV, acute angle between DGCV and AIV, the thin lumen of Summit-CV, are all potential anatomic factors preventing catheter ablation of DGCV-AIV VAs. Therefore, any method, which could assist ablation catheter overcoming these anatomic obstacles, would contribute to the successful ablation of DGCV-AIV VAs Figure 2.

### Follow-Up

Each patient returned for evaluation in the hospital's outpatient department of cardiology 1-month after treatment. Twelve-lead ECG and 24-h Holter monitoring were performed at the 3-month follow-up visit. All patients received coronary CT angiography using 128-slice dual-source CT 3-months later to detect the long-term effect of ablation in DGCV-AIV to an adjacent coronary artery. Meanwhile, the anatomy of the coronary venous system was evaluated in each case using maximum intensity projection and volume rendering technique multi-planar reformation reconstructions. The angle between AIV and DGCV, and width/ height of GCV in each patient was measured by three radiologists independently, a mean value was adopted for statistical analysis. The height of GCV was defined as the maximal vertical distance from the beginning of AIV to the proximal GCV. The width of GCV was defined as the maximal transversal distance from lateral GCV to the maximal vertical line.

### Statistical Analysis

Continuous variables are expressed as mean ± SD. Continuous variables were compared using a *t*-test if a normal distribution was assumed or using a Mann-Whitney U test if a normal distribution was not assumed. Categorical variables were compared using the χ^2^-test. A 2-tailed *P* < 0.05 was considered significant.

## Results

**Figure 3:**
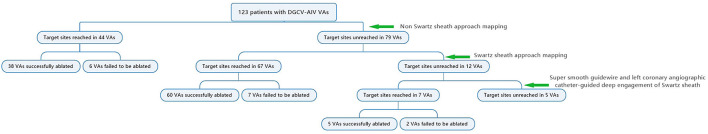
Flowchart of mapping and ablation procedure in this study.

**Figure 4:**
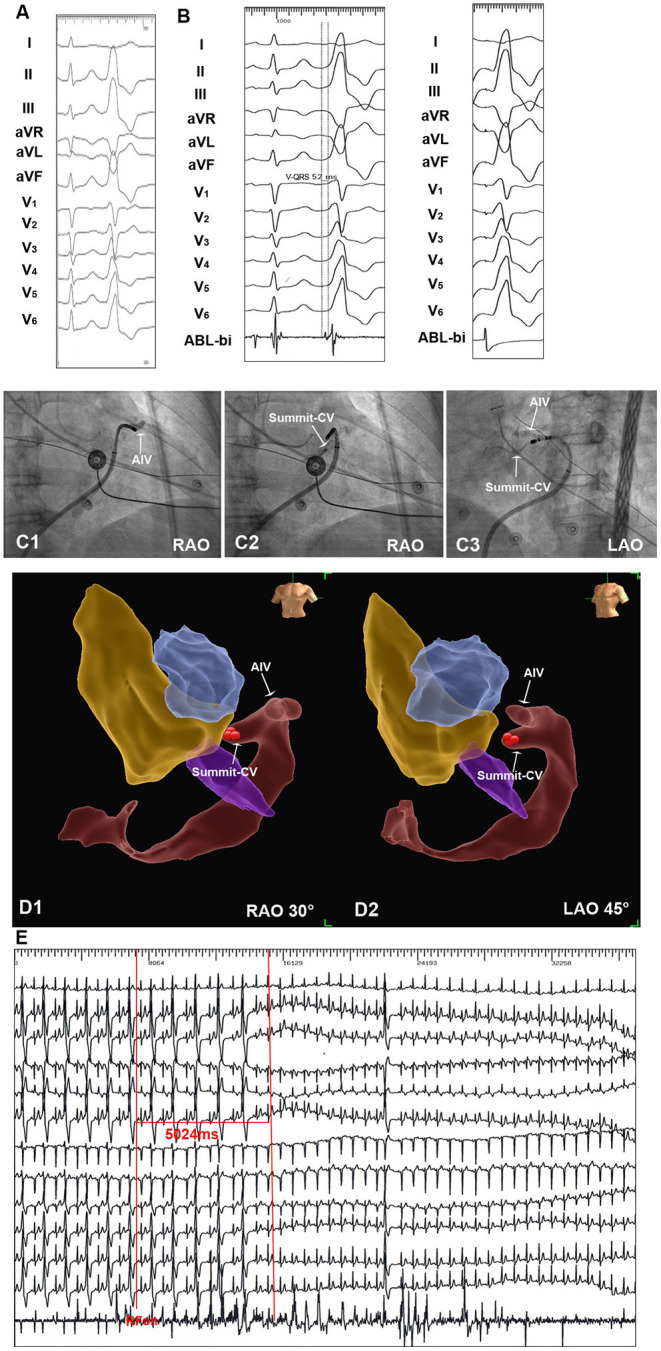
An example of successful ablation of premature ventricular complex (PVC) originating from Summit-CV by SS approach. **(A)** Twelve-lead ECG 3 morphology of the clinical PVC. The PVC showed R wave in II, III, aVF, V4-V6, rS morphology in lead V1, the precordial transition zone of PVC was earlier than sinus beat. **(B)** By NS approach, the irrigated catheter could reach the distal portion of GCV. SS approach was then applied in this patient. By SS approach, the irrigated catheter reached the target site in summit-CV. The local ventricular activation time recorded at the summit-CV preceded the onset of the QRS complex by 52 ms, and a perfect pace-match was achieved by pacing at summit-CV. **(C1–C3)** Fluoroscopic view of the target site in summit-CV. **(D1,D2)** Three-dimension view of target site in Summit-CV. **(E)** Radiofrequency energy delivered on target site for 5 seconds led to an acute disappearance of target PVCs.

The 123 consecutive cases of DGCV VAs undergoing mapping and ablation in our center were retrospectively reviewed in this study, shown in Figure 3. NS approach and SS approaches were attempted in 123 and 79 cases, respectively. By NS approach, DGCV-AIV target site reaching was only obtained in 44 VAs (30.89%, 44/123) with successful ablation in 38 VAs (30.89%, 38/123) VAs. Via SS approach, DGCV-AIV target site reaching was obtained in 67 VAs (84.81%, 67/79) with successful ablation in 60 VAs (75.95%, 60/79). In 12 VAs, target sites failed to be reached by both NS approach and SS approach, the hydrophilic coated guide wire, and left coronary angiographic catheter-guided deep engagement of Swartz sheath in GCV to support ablation catheter was applied. In this way, the irrigated catheter was delivered to distal sites of DGCV-AIV. Among these 12 VAs, target sites were achieved in 7 VAs (58.33%, 7/12) with successful ablation in 5 cases (41.67%, 5/12). There were no significant differences in catheter tip reaching coronary sinus, proximal GCV, or middle GCV by NS approach and SS approach. Of note, some distal sites of GCV (DGCV1, DGCV2, AIV, Summit-CV) could be more possibly reached by catheter tip via SS approach, shown in Table 1. A successful ablation case of DGCV-AIV VAs by SS approach post failed NS approach was shown in Figure 4.

**Table 1:** Comparison of Catheter tip reaching sites and success rate between two manipulation methods (Cases, %).

	**Group**		
	**NS approach (*n* = 123)**	**SS approach (*n* = 79)**	**-Value**
Coronary sinus	123 (100.00%)	79 (100.00%)	>0.05
Proximal GCV	114 (92.68%)	74 (93.67%)	>0.05
Middle GCV	110 (93.50%)	72 (91.14%)	>0.05
DGCV1	49 (39.84%)	70 (88.61%)	<0.00
DGCV2	43 (34.96%)	69 (87.34%)	<0.00
AIV	21 (17.07%)	30 (37.97%)	<0.00
Summit-CV	2 (1.63%)	7 (8.86%)	<0.05
Reaching target sites	44 (35.77%)	67 (84.81%)	<0.00
Successful ablation	38 (30.89%)	60 (75.75%)	<0.00

Due to the obstacle of venous valves of CVS, middle GCV could not be reached in 13 VAs and in 7 VAs by NS approach and SS approach, respectively. Nevertheless, via the hydrophilic coated guidewire and left coronary angiographic catheter-guided deep engagement of Swartz sheath in GCV, the obstacle of the venous valve was overcome. As the guidewire went through the venous valves smoothly, the angiographic catheter advanced to the distal portion of CVS along the guide wire, which provided a backup force for the Swartz sheath and facilitate the Swartz sheath to reach the middle GCV.

By NS approach, the catheter tip accessed DGCV2 in 43 patients, among which, catheter tip could achieve AIV in only 21 patients. Via SS approach, catheter tip accessed DGCV2 in 69 patients, and the catheter tip could further achieve AIV in 30 patients. CTV of CVS 3-month post-RFCA compared the patients of AIV reached by catheter tip with the patients of AIV not reached (51 VAs: 72.05 ± 14.62° vs. 61 VAs: 108.73 ± 17.61°). The angle between AIV and DGCV2 ≤83° had a sensitivity of 94.1%, specificity of 77.0%, and accuracy of 94.5% for identifying the inaccessibility from DGCV2 to AIV, no matter SS approach or NS approach used, shown in Figure 2.

Whether the CVS morphology would affect the catheter manipulation approach selected was also investigated. In 44 VAs with target sites reached by the NS approach, a smaller Width/Height of CVS was more found. On the contrary, in 67 VAs with target sites reached by the SS approach, a relatively larger Width/Height of CVS was observed. A W/H of CVS>0.69 had a sensitivity of 91.0%, specificity of 68.2%, and accuracy of 87% for identifying a SS approach application, shown in Figure 2, Table 2.

**Table 2:** Reasons for failure of reaching target sites (Cases,%).

	**Group**		
	**NS approach (*n* =123)**	**SS approach (*n* =79)**	***P*-Value**
Obstacle of Venous valves	13	7	>0.05
Narrow angle between DGCV2 and AIV (>83°)	22/43	33/69	>0.05
Failure to reach Summit-CV	121	72	<0.05
Width/Height ratio>0.69	14/44	60/67	<0.05

### Electrophysiological Mapping and Ablation

A series of mapping and ablation parameters of successful ablated DGCV-AIV VAs by NS approach and by SS approach were also compared. There were no significant differences in the local ventricular activation time relative to the QRS onset (V-QRS), ventricular capture ratio, pace-match leads, procedure time, RF duration, number of RF lesions, and fluoroscopic time. The operation time in CVS by SS approach was slightly longer than by NS approach, shown in Table 3.

**Table 3:** Comparison of electrophysiological study and radiofrequency catheter ablation of successful ablated VAs between two manipulation methods.

	**Group**		
	**NS approach (*n* = 38)**	**SS approach (*n* = 60)**	***P*-Value**
V-QRS, ms	−34.52 ± 6.73	−34.87 ± 5.66	>0.05
Ventricular capture	32 (84.21%)	55 (84.61%)	>0.05
Pace match leads	11.22 ± 1.34	11.40 ± 1.65	>0.05
Procedure time, min	64.18 ± 12.64	67.11 ± 15.09	>0.05
Operation in CS, min	32.63 ± 5.27	40.72 ± 4.43	<0.05
RF duration, s	147.65 ± 58.61	142.03 ± 61.37	>0.05
No. of RF lesions	1.74 ± 0.58	1.77 ± 0.64	>0.05
Fluoroscopic time, min	10.98 ± 4.98	11.98 ± 5.33	>0.05

Complications during the procedure by the NS approach and the SS approach were also compared. There were no significant differences in complications between these two groups (4/123, 3.25% vs. 7/79, 8.86%, *p* > 0.05). Via SS approach, coronary vein dissection happened on three patients and coronary vein rupture happened on two patients. The two patients of coronary vein rupture developed cardiac tamponade but turned into hemodynamic stability post-emergent pericardiocentesis. One patient has severe chest pain with CAG showing an acute irreversible 50% coronary stenosis in LAD, another patient had an episode of chest tightness, and coronary angiography revealed coronary spasm of LCx, relieved by intravenous nitroglycerin. By NS approach, coronary vein dissection happened on two patients and coronary vein rupture occurred on one patient. Coronary vein rupture caused delayed pericardial effusion but without hemodynamic instability, thus pericardiocentesis was not performed. Coronary spasm occurred on one patient but was relieved by intravenous nitroglycerin, shown in Table 4.

**Table 4:** Comparison of complications between these two manipulation methods (Cases,%).

	**Group**		
	**NS approach (*n* = 123)**	**SS approach (*n* = 79)**	***P*-Value**
Coronary vein dissection	2	3	>0.05
Coronary vein rupture	1^*^	2^#^	>0.05
Cardiac tamponade	0	2^#^	>0.05
Delayed pericardial effusion	1^*^	0	>0.05
Coronary artery injury	1	1	>0.05
Coronary artery spasm	0	1	>0.05
Death	0	0	>0.05
Total complications	4 (3.25%)	7 (8.86%)	>0.05

* *the same patients in the manipulation without long sheath group*;

# *the same patients in the manipulation with long sheath group*.

## Discussion

### Major Findings

This study reports for the first time that VAs arising from DGCV-AIV can be mapped and ablated by the Swartz sheath support approach with high efficiency and success rate compared to the non-Swartz sheath support approach. Width/height of coronary venous system>0.69 favored a SS approach. However, an angle between DGCV and AIV <83° indicated an inaccessible AIV by ablation catheter.

### RFCA VAs Arising From DGCV-AIV

A total of 2768 VAs received RFCA in our cardiac lab, and 4.44% VAs (123/2768) were found arising from the region of DGCV-AIV. Successful ablation was achieved in 102 patients (102/123). As is well-known, DGCV-AIV is the epicardial part of LVOT, the myocardium near the DGCV-AIV can be a source of idiopathic VAs. Yamada T et al. studied 27 consecutive patients with VAs originating from the epicardial LVOT and achieved successful ablation within the DGCV in 14 patients (5). Hachiya H et al. also reported successful catheter ablation of idiopathic VAs originating from the AIV (6). More recently, Yuki K et al. reported that 14 patients were found to have summit-CV VAs and successful ablation was achieved in 10 (71%) patients (7). Therefore, VAs arising from DGCV-AIV were not a rare phenomenon and catheter ablation is an effective treatment for DGCV-AIV VAs.

### Catheter Ablation Approach for DGCV-AIV VAs

Previous studies have revealed that one key point for successful ablation of DGCV-AIV VAs is the structure of DGCV-AIV being sufficiently accessed and mapped. However, the existence of anatomic obstacles in the coronary venous system limited the ablation catheter manipulation and access to the target sites in this region. In some cases, even advancing the ablation catheter to the proximal GCV is difficult. In our study, due to the obstacle of venous valves, the catheter tip could not reach the proximal-middle GCV by NS approach and SS approach in seven patients. One study reported in one patient with DGCV VAs, because of the tortuous course of GCV, catheter ablation could not access the optimal target site of VAs (8). The deflectable sheath and contact force ablation catheter was then advanced alternately, overcoming the deflection of GCV, leading to a deep engagement of catheter tip to DGCV, and achieving successful ablation consequently. However, this method had its limitations and might not be widely applied to most patients. In patients with a coronary venous system smaller in size, it could be challenging to manipulate the contact force catheter and steerable sheath, as both of which were much larger than the conventional irrigated catheter and sheath in diameter. Besides, the much harder characteristics of steerable sheath and contact force catheter may more easily cause coronary vein dissection and rupture. Another study also reported anatomic obstacles that restrained successful ablation of DGCV-AIV VAs (7). Due to the very distal portion of DGCV are usually very thin and frequently inaccessible to an ablation catheter, Kazutaka A et al. delivered a 2F microcatheter into the vein as a landmark of the ablation sites and performed ablation in the nearby endocardial structures. However, by this approach, the elimination of these VAs usually requires ablation at multiple sites at adjacent structures and because of indirect ablation, the efficacy of RFCA was usually limited and more complications unpredictable. In our research, we detected that in a situation of a narrow elliptical shape of CVS (Width/Height>0.69), SS approach ensured a powerful backup force for ablation catheter, which could assist the catheter tip in overcoming partial anatomic impediments of the coronary venous system and reach the target sites in DGCV-AIV more easily, contributing to a relatively higher success rate of RFCA. Thus, it was more favorable than the NS approach when RFCA of DGCV-AIV VAs. Nonetheless, we found an acute angle between AIV and DGCV was in fact an anatomic obstacle difficult to overcome no matter whether an NS approach or SS approach was applied, for which, the appropriate manipulation approach remains to be investigated. In addition, It should be noted that when catheter ablation of DGCV-AIV VAs, anatomic obstacles are not the only factors that restrain successful ablation. In clinical practice, successful ablation of DGCV-AIV VAs is associated with a network of factors, including origin sites (epicardial but not intramural), distance to adjacent coronary artery (distal but not proximal), impedance (not too high to limit energy delivery) during ablation and so on. In our study, failed ablation happened on 15 patients with good target sites accessed by catheter tip. The reasons underlying the failure are mostly the factors mentioned above.

### Complications of Swartz Sheath Support Approach for DGCV-AIV VAs

Of note, a relatively higher rate of cardiac vein dissection and rupture was observed in the SS approach. We speculated that while the SS approach application provided a better backup for catheter manipulation, it also increased the contact force of the catheter tip to the coronary vein, contributing to a higher incidence of coronary vein damage.

In our study, coronary injury was found in both groups. Though the safe distance from the catheter tip to the adjacent coronary artery has been demonstrated by coronary angiography, coronary injury is still a potential complication, which could not be neglected.

### Study Limitations

As the study was retrospective research, results need to be confirmed by prospective studies. Further studies with multi-center cooperation and a larger sample size are needed to confirm the findings.

## Conclusion

VAs arising from DGCV-AIV is not a rare phenomenon. For catheter ablation of DGCV-AIV VAs, the Swartz sheath support approach facilitates the access of target sites and improves the success rate of RFCA.

## Data Availability Statement

The original contributions presented in the study are included in the article/supplementary material, further inquiries can be directed to the corresponding authors.

## Ethics Statement

The studies involving human participants were reviewed and approved by Second Affiliated Hospital and Yuying Children's Hospital's Ethics Committee. The patients/participants provided their written informed consent to participate in this study. Written informed consent was obtained from the individual(s) for the publication of any potentially identifiable images or data included in this article.

## Author Contributions

CZ and J-FL designed and drafted the original research. CZ, W-QL, Y-JW, Q-QJ, F-ZL, JL, and J-FL performed the follow-up and data analysis for the study. All authors approved it for publication.

## Funding

This work was supported by the Wenzhou Municipal Science and Technology Commission (grant no. ZY2020018), the Zhejiang Provincial Natural Science Foundation (grant no. LY21H020011), and the National Natural Science Foundation of China (grant no. 82070333). The funders had no role in study design, data collection, and analysis, decision to publish, or preparation of the manuscript.

## Conflict of Interest

The authors declare that the research was conducted in the absence of any commercial or financial relationships that could be construed as a potential conflict of interest.

## Publisher's Note

All claims expressed in this article are solely those of the authors and do not necessarily represent those of their affiliated organizations, or those of the publisher, the editors and the reviewers. Any product that may be evaluated in this article, or claim that may be made by its manufacturer, is not guaranteed or endorsed by the publisher.

## References

[1] Habib A Lachman N Christensen KN Asirvatham SJ. The anatomy of the coronary sinus venous system for the cardiac electrophysiologist. Europace. (2009) 11(Suppl. 5):v15–21. 10.1093/europace/eup270 19861386

[2] Chen YH Lin JF. Catheter ablation of idiopathic epicardial ventricular arrhythmias originating from the vicinity of the coronary sinus system. J Cardiovasc Electrophysiol. (2015) 26:1160–7. 10.1111/jce.12756 26175213

[3] Liao Z Zhan X Wu S Xue Y Fang X Liao H. Idiopathic ventricular arrhythmias originating from the pulmonary sinus cusp: prevalence, electrocardiographic/electrophysiological characteristics, and catheter ablation. J Am Coll Cardiol. (2015) 66:2633–44. 10.1016/j.jacc.2015.09.094 26670064

[4] Ouyang F Mathew S Wu S Kamioka M Metzner A Xue Y. Ventricular arrhythmias arising from the left ventricular outflow tract below the aortic sinus cusps: mapping and catheter ablation via transseptal approach and electrocardiographic characteristics. Circ Arrhythm Electrophysiol. (2014) 7:445–55. 10.1161/CIRCEP.114.001690 24795340

[5] Yamada T McElderry HT Doppalapudi H Okada T Murakami Y Yoshida Y. Idiopathic ventricular arrhythmias originating from the left ventricular summit: anatomic concepts relevant to ablation. Circ Arrhythm Electrophysiol. (2010) 3:616–23. 10.1161/CIRCEP.110.939744 20855374

[6] Hachiya H Hirao K Nakamura H Taniguchi H Miyazaki S Komatsu Y. Electrocardiographic characteristics differentiating epicardial outflow tract-ventricular arrhythmias originating from the anterior interventricular vein and distal great cardiac vein. Circ J. (2015) 79:2335–44. 10.1253/circj.CJ-15-0476 26346171

[7] Komatsu Y Nogami A Shinoda Y Masuda K Machino T Kuroki K. Idiopathic ventricular arrhythmias originating from the vicinity of the communicating vein of cardiac venous systems at the left ventricular summit. Circ Arrhythm Electrophysiol. (2018) 11:e005386. 10.1161/CIRCEP.117.005386 29326128

[8] Kumagai YU Arimoto T Iwayama T Hashimoto N Watanabe T Kubota I. Contact force-guided deep engagement with a steerable sheath in the distal great cardiac vein: a case report. Pacing Clin Electrophysiol. (2016) 39:507–10. 10.1111/pace.12826 26854279

